# A Ru-Complex Tethered to a N-Rich Covalent Triazine Framework for Tandem Aerobic Oxidation-Knoevenagel Condensation Reactions

**DOI:** 10.3390/molecules26040838

**Published:** 2021-02-05

**Authors:** Geert Watson, Parviz Gohari Derakhshandeh, Sara Abednatanzi, Johannes Schmidt, Karen Leus, Pascal Van Der Voort

**Affiliations:** 1 Department of Chemistry, Center for Ordered Materials, Organometallics and Catalysis (COMOC), Ghent University, Krijgslaan 281, Building S3 (Campus Sterre), 9000 Gent, Belgium; geert.watson@ugent.be (G.W.); parviz.gohariderakhshandeh@ugent.be (P.G.D.); Karen.Leus@ugent.be (K.L.); 2 Institut für Chemie-Funktionsmaterialien, Technische Universität Berlin, Hardenbergstraße 40, 10623 Berlin, Germany; johannes.schmidt@tu-berlin.de

**Keywords:** covalent triazine frameworks, heterogeneous catalysis, tandem catalysis, aerobic oxidation-knoevenagel condensation

## Abstract

Herein, a highly N-rich covalent triazine framework (CTF) is applied as support for a Ru^III^ complex. The bipyridine sites within the CTF provide excellent anchoring points for the [Ru(acac)_2_(CH_3_CN)_2_]PF_6_ complex. The obtained robust Ru^III^@bipy-CTF material was applied for the selective tandem aerobic oxidation-Knoevenagel condensation reaction. The presented system shows a high catalytic performance (>80% conversion of alcohols to α, β-unsaturated nitriles) without the use of expensive noble metals. The bipy-CTF not only acts as the catalyst support but also provides the active sites for both aerobic oxidation and Knoevenagel condensation reactions. This work highlights a new perspective for the development of highly efficient and robust heterogeneous catalysts applying CTFs for cascade catalysis.

## 1. Introduction

Conventional porous materials including silica, zeolite and activated charcoal have attracted extensive interest in large-scale industrial applications, most importantly in heterogeneous catalysis [1,2,3]. However, the poor chemical versatility of their chemical structure has increased the need for alternative porous materials with tailorable properties. In recent years, the focus in heterogeneous catalysis lies in the development of novel and efficient porous supports with tailor-made functionalities rather than prefabricated materials for targeted liquid phase reactions [4,5]. The recently emerging porous materials, particularly metal–organic frameworks (MOFs) and covalent organic frameworks (COFs) have led to excellent progress in the field [6]. These materials possess a large surface area with regular and accessible pores, and an adjustable skeleton, making them attractive for several purposes of interest [7]. In contrast to MOFs fabricated from inorganic nodes (metal ions/clusters), COFs are purely organic materials and constructed from covalently linking light atoms (C, O, N, P, B, Si) [8,9]. Covalent Triazine Frameworks (CTFs) are a class of COFs discovered in 2008 by Thomas and Antonietti [10]. CTFs are formed through a trimerization reaction followed by the subsequent oligomerization of aromatic nitriles [11]. The robust aromatic covalent bonds endow CTFs with excellent stability compared to coordinative-linked porous materials [12]. Additionally, CTFs contain a high amount of nitrogen functionalities in their networks, allowing them to be outstanding candidates as supports for various catalytic active centers [13].

Among a wide range of catalytic processes, tandem catalysis has attracted increasing research attention [14]. In tandem catalysis, several consecutive catalytic reactions occur consecutively in one reaction vessel, using only one multifunctional catalyst. Therefore, there is no need for the separation, purification, and transfer of intermediates produced in each step. Tandem catalysis significantly reduces the amount of waste and minimizes the use of harmful solvents [15]. Great efforts have been made to design heterogeneous catalysts for tandem reactions through the immobilization of metal complexes and nanoparticles on the surface of various porous supports [14,16]. α, β-unsaturated nitriles are key intermediates for the synthesis of pharmaceuticals and fine chemicals [17]. These intermediates are generally produced through the Knoevenagel condensation of aldehydes or ketones with nitriles catalyzed by common bases [18]. However, the catalytic process suffers from limited substrate scope due to the high price or unavailability of some aldehydes [18]. In this regard, the development of highly efficient multifunctional catalysts to prepare α, β-unsaturated nitriles through the tandem aerobic oxidation-Knoevenagel condensation reaction significantly boosts the synthesis efficiency.

Highly efficient oxidation catalysts for the selective conversion of alcohols to aldehydes are a key step in designing an appropriate heterogeneous catalyst for the tandem oxidation-Knoevenagel condensation reaction. Traditional oxidation processes employ stoichiometric amounts of sometimes toxic and expensive inorganic oxidants, mainly iodosylbenzene, sodium hypochlorite and chromium trioxide [19,20,21,22].

Many papers have appeared on the design of various homogeneous and heterogeneous catalysts containing noble metals, such as Au, Pd, Pt and Ir, for the selective aerobic oxidation of alcohols [23,24,25,26]. Ru catalysts are economically attractive in comparison to other noble-metal catalysts, which are rather expensive. Ru^III^ complexes are well documented as efficient oxidation catalysts for various substrates, such as alcohols, aldehydes and sulfides. Nevertheless, the majority of studies using ruthenium, either homo- or heterogeneously, utilize non-green oxidants (3-dichloroiodanyl-benzoic acid, periodic acid and iodosylbenzene) [19,27,28].

Recently, our group reported on the immobilization of a Ru^III^ complex onto a periodic mesoporous organosilica (PMO) [29]. Although the catalyst was highly active for the selective oxidation of alcohols using periodic acid, no activity was observed using oxygen as the green oxidant. To date, only a limited amount of studies have been reported on the application of Ru^III^-based catalysts in the aerobic oxidation of alcohols [30,31,32]. Moreover, many of these catalysts exhibit fundamental drawbacks as high catalyst loadings (5 mol% [Ru]) or a large excess of oxygen (20 atm) is required as the oxidant [33,34]. Thus, the development of greener and more atom-efficient methods that adopt recyclable catalysts and molecular oxygen as the sole oxidant is a great alternative to the existing systems.

We introduce here an efficient catalytic system for the tandem aerobic oxidation-Knoevenagel condensation reaction. A highly N-rich CTF containing bipyridine (bipy) building blocks (bipy-CTF) is used as the catalyst support. The bipy building units provide excellent docking sites for immobilization of a Ru^III^ complex, initially examined in the selective aerobic oxidation of alcohols to aldehydes. The bipy-CTF material not only acts as an anchoring point but also promotes the sequential reaction of aldehydes and nitriles due to the presence of N-rich basic functionalities. Our results indicate that the synergistic effects between the N-rich bipy-CTF and the Ru^III^ complex are beneficial to obtaining a highly active and selective catalyst for tandem catalysis in the absence of any co-oxidant.

## 2. Results and Discussion

### 2.1. Synthesis and Characterization of the Modified Bipy-CTF with the Ru Complex (Ru^III^@bipy-CTF)

**Figure 1:**
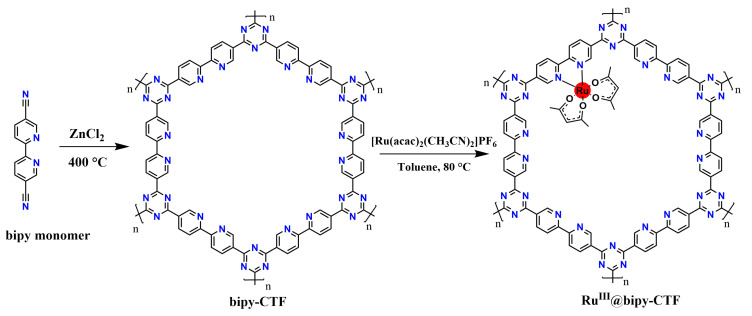
Schematic representation of the ideal ordered structure of Ru^III^@bipy-covalent triazine framework (CTF) material.

We targeted a CTF with free 2,2′-bipyridine building blocks (5,5′-dicyano-2,2′-bipyridine), which forms excellent anchoring points. The bipy-CTF material was synthesized following the typical reported ionothermal procedure [35]. After the synthesis, the remaining ZnCl_2_ is removed by extensive washing with water, followed by refluxing at 120 °C in 1 M HCl. The obtained bipy-CTF material was post-modified with the (Ru(acac)_2_(CH_3_CN)_2_)PF_6_ complex through a simple wet impregnation method, as depicted in Figure 1.

**Figure 2:**
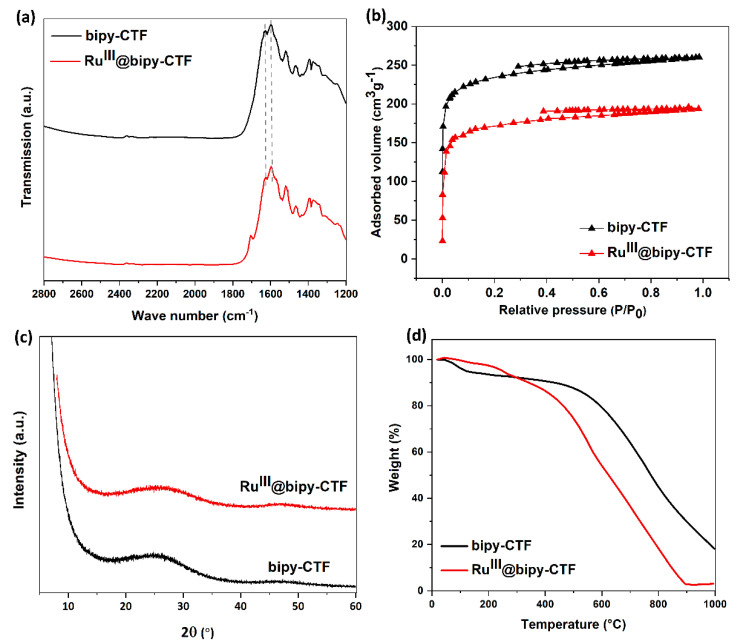
Structural characterization of bipy-CTF and Ru^III^@bipy-CTF materials. (**a**) Diffuse reflectance infrared Fourier transform (DRIFT) spectra. (**b**) Nitrogen adsorption/desorption isotherms. (**c**) Powder X-ray diffraction (XRD) patterns. (**d**) Thermogravimetric analysis (TGA) curves.

In the diffuse reflectance infrared Fourier transform (DRIFT) spectrum of the bipy-CTF (Figure 2a), the characteristic bands of the triazine fragment appear at 1356 and 1521 cm^−1^. The absence of the intense nitrile band at around 2330 cm^−1^ demonstrates the complete consumption of monomer and formation of triazine linkages. The doublet band at around 1602–1626 cm^−1^ is ascribed to the C=N vibrations of the bipy moiety. In the DRIFT spectrum of the Ru^III^@bipy-CTF material, the vibration bands of the bipy moiety are shifted (~10 cm^−1^) to a lower frequency, which may be due to coordination with the Ru complex. A similar observation is reported in previous studies [36].

The pristine bipy-CTF material displays a rapid uptake of N_2_ at low relative pressures which is indicative of a highly microporous material (Figure 2b). This profile is assigned to a type I isotherm and exhibits a Brunauer–Emmett–Teller (BET) surface area of 787 m^2^ g^−1^. The total pore volume was found to be 0.40 cm^3^ g^−1^ at P/P_0_ = 0.99. After the introduction of the Ru complex, the BET surface area decreases moderately to 556 m^2^ g^−1^, indicating that most of the pores are still accessible.

The powder X-ray diffraction (PXRD) patterns of the pristine bipy-CTF and Ru^III^@bipy-CTF are shown in Figure 2c. As known from most of the CTFs that were prepared ionothermally, the bipy-CTF materials were found to be predominantly amorphous. The broad peaks at 2θ~13 and 25° are assigned to the 00l reflection showing a ‘‘graphitic’’ layer stacking. It is important to note that the exact structure of these amorphous materials cannot be determined since the harsh synthesis conditions result in carbonization and blackening of the material, making it difficult to fully characterize. To estimate the carbonization degree and purity of the materials, we applied elemental analysis (Table 1). The CHN data obtained from the bipy-CTF material reveal a C/N ratio of 2.9 and the theoretical value for C/N in the bipy-CTF sample is calculated to be 2.6. Therefore, partial carbonization of around 10% occurs, while 90% of the structural composition is preserved.

The thermal stability of both materials was determined by thermogravimetric analysis (TGA). The TGA profile of the bipy-CTF displayed a high thermal stability up to approximately 550 °C (Figure 2d). A first weight loss of about 8% below 150 °C corresponds to the loss of water and organic solvent molecules. The TGA profile of the modified sample shows that the Ru^III^@bipy-CTF material is thermally stable up to 300 °C, and gradually decomposes at higher temperatures.

**Figure 3:**
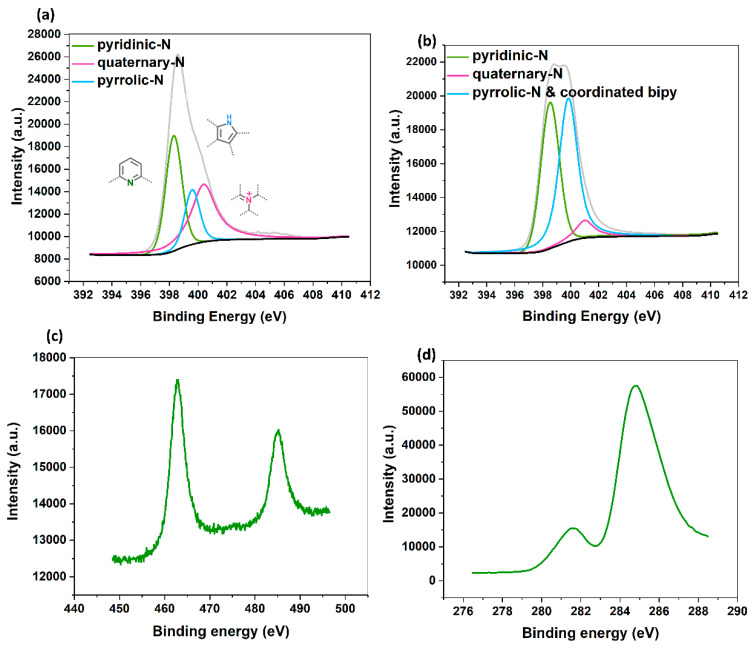
Structural characterization of bipy-CTF and Ru^III^@bipy-CTF materials. (**a**) N 1S XPS spectrum of the bipy-CTF. (**b**) N 1s XPS spectrum of the Ru^III^@bipy-CTF. (**c**) Ru 3p XPS spectrum of the Ru^III^@bipy-CTF. (**d**) Ru 3d XPS spectrum of the Ru^III^@bipy-CTF.

Further structural characterization was done by applying X-ray photoelectron spectroscopy (XPS). In the N 1S spectrum of the bipy-CTF material (Figure 3a), a peak at 398.39 eV confirms the existence of the pyridinic nitrogen in the framework. Besides, the peaks at 399.58 and 400.38 eV are attributed to the pyrrolic-and quaternary-N species, respectively. These nitrogen functionalities are formed during the synthesis at high temperatures, as reported by Osadchii et al. [37]. In the N 1s spectrum of the Ru^III^@bipy-CTF material (Figure 3b), a peak shift towards a higher binding energy is observed for the pyridinic N species which overlaps with the peak of pyrrolic-N sites. Such a N 1s shift towards higher binding energies can be attributed to the slight transfer of electrons to the immobilized Ru complexes [38]. The Ru 3p peaks for the Ru^III^@bipy-CTF are located at around 463 and 485 eV, which corresponds to Ru in the (+3) oxidation state (Figure 3c). Moreover, the Ru 3d peak is seen at 285 eV (Figure 3d). Based on the inductively coupled plasma (ICP) analysis, the loading of Ru in the modified material is 0.15 mmol g^−1^, and around 3% of the total bipyridine sites are coordinated to the Ru complex.

### 2.2. Catalytic Activity of the Ru^III^@bipy-CTF Catalyst in the Tandem Aerobic oxidation-Knoevenagel Condensation Reaction

The catalytic activity of heterogeneous Ru catalysts for oxidation reactions using O_2_ or air as the green oxidant remains a challenge. Initially, the catalytic activity of the Ru^III^@bipy-CTF catalyst was tested under aerobic conditions for the selective oxidation of benzyl alcohol to benzaldehyde. The catalytic results are presented in Table 2. The Ru^III^@bipy-CTF catalyst displays a moderate activity with a conversion of 37% using toluene as the reaction medium (Table 2, entry 1). To further enhance the catalytic performance of the catalyst, different bases were applied (Table 2, entries 2–4). The catalytic conversion of benzyl alcohol increases in the presence of K_2_CO_3_ and Cs_2_CO_3_ with conversions of 78 and 99%, respectively. Notably, no product of over-oxidation (benzoic acid) was detected, proving the high selectivity of the Ru catalyst towards benzaldehyde. In the absence of the catalyst, no conversion of benzyl alcohol was observed (<1% after 12 h). Moreover, the conversion of benzyl alcohol toward benzaldehyde decreased to 3% under an Ar atmosphere, which confirms the essential need for oxygen as the oxidant (entry 7 in Table 2). The catalytic activity of the Ru^III^@bipy-CTF catalyst was further compared with its homogeneous counterpart (Table 2, entry 8). Under the same reaction conditions, the (Ru(acac)_2_(CH_3_CN)_2_)PF_6_ complex showed lower catalytic conversion (54% using Cs_2_CO_3_). The improved activity of the Ru^III^@bipy-CTF catalyst can be attributed to the contributing role of the bipy-CTF support (see mechanistic studies in the Supplementary Materials, Table S1 and Figure S1). A control experiment was performed using the pristine bipy-CTF material. A conversion of 39% was obtained using the pristine CTF as the catalyst under identical reaction conditions (Table 2, entry 9). It has been proven that nitrogen-rich carbon materials are effective catalysts for aerobic oxidation reactions [39]. We recently showed the unique properties of CTFs to proceed with aerobic oxidation reactions assisted by nitrogen functionalities [40,41]. More specifically, CTFs with quaternary N species can activate molecular O_2_ to generate oxygen radicals (superoxide) which further promote the oxidation reaction.

Our further studies focused on the catalytic performance of the Ru^III^@bipy-CTF catalyst in the tandem aerobic oxidation-Knoevenagel condensation reaction. For this purpose, the optimized reaction condition for the oxidation of benzyl alcohol was selected (1 mol% Ru^III^@bipy-CTF, Cs_2_CO_3_, O_2_, 100 °C, 12 h). Different substituted benzyl alcohols and malononitrile were examined and the obtained results are listed in Table 3. No product was formed in the absence of the catalyst. As shown in Table 3, the Ru^III^@bipy-CTF catalyst was found to be highly active in the Knoevenagel condensation reaction under mild reaction conditions. A high conversion was obtained for all substrates at a low temperature (70 °C) and only after 1 h. Moreover, complete selectivity was observed towards the corresponding product.

**Figure 4:**
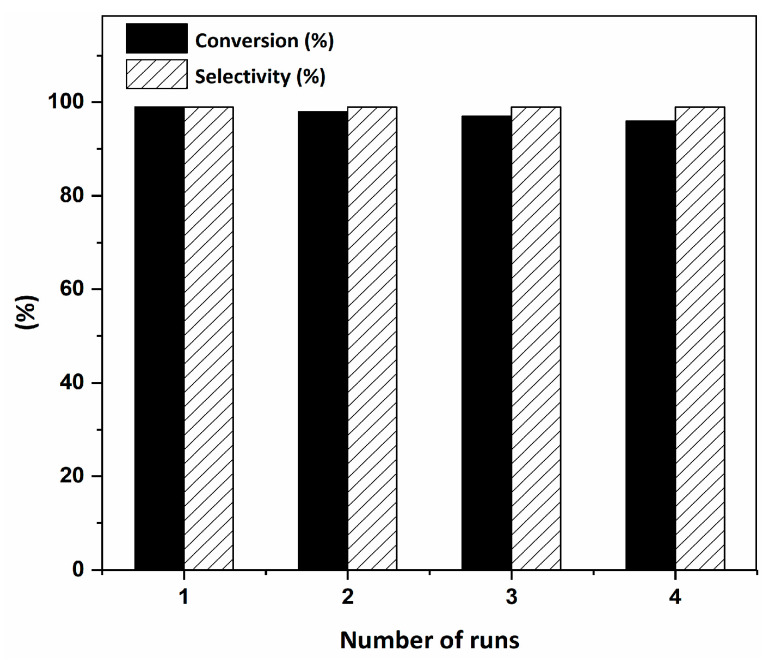
Recyclability of the Ru^III^@bipy-CTF catalyst (1 mol% catalyst, 0.33 mmol benzyl alcohol, 0.4 mmol Cs_2_CO_3_, 500 μL toluene, 0.33 mmol malononitrile, O_2_, 100 °C, 12 h (1st step) and 70 °C, 1 h (2nd step).

The recyclability of the Ru^III^@bipy-CTF catalyst was investigated for the tandem aerobic oxidation-Knoevenagel condensation reaction. The recyclability studies showed that the Ru^III^@bipy-CTF catalyst maintains almost its full catalytic performance after four consecutive cycles with no obvious loss of activity or selectivity (Figure 4). Moreover, the recycled catalyst showed no detectable Ru leaching (analyzed by ICP-OES).

A comparison of the catalytic performance of the presented system is made with various catalysts for the tandem aerobic oxidation-Knoevenagel condensation reaction. It is challenging to make a fair comparison since, in almost all the studies, no turnover number (TON) or turnover frequency (TOF) values were reported. Therefore, a comparison can only be made in terms of conversion to provide an overall overview. As can be seen from Table 4, a high yield of benzylmalononitrile is obtained over the Ru^III^@bipy-CTF catalyst using O_2_ as the green oxidant and in the absence of any co-oxidant. Moreover, the present system has the advantage of a high catalytic performance without the use of expensive noble metals.

## 3. Materials and Methods

### 3.1. Materials and Instrumentation

X-ray powder diffraction (XRPD) patterns were collected on a Thermo Scientific ARL X’Tra diffractometer, operated at 40 kV, 40 mA, using Cu−Kα radiation (λ = 1.5406 Å). Nitrogen sorption studies were performed at −196 °C using a Belsorp-mini II gas analyzer. Before the adsorption experiments, the samples were degassed under vacuum at 120 °C to remove adsorbed water. An ultra-fast GC equipped with a flame ionization detector (FID) and a 5% diphenyl/95% polydimethylsiloxane column, with 10 m length and 0.10 mm internal diameter, was used to follow the conversion of the products during the catalytic tests. Helium was used as the carrier gas and the flow rate was programmed as 0.8 mL/min. The reaction products were identified with a TRACE GC × GC (Thermo, Interscience, Waltham, MA, USA), coupled to a TEMPUS TOF-MS detector (Thermo, Interscience, Waltham, MA, USA). Thermogravimetric analysis (TGA) was carried out to determine the stability of the CTF materials using a NET-ZSCH STA 409 PC/PGTG instrument. The samples were heated from 30 to 1000 °C in air at a constant rate of 10 °C/min. The X-ray photoelectron spectroscopy (XPS) measurements were performed on a K-alpha Thermo Fisher Scientific spectrometer with a monochromatic Al Kα X-ray source. Metal content was determined by an ICP-OES Optima 8000 (inductively coupled plasma optical emission spectroscopy) atomic emission spectrometer. The nitrogen content of the materials was determined with a Thermo Flash 200 elemental analyzer using V_2_O_5_ as the catalyst.

All chemicals were purchased from Sigma-Aldrich, abcr or TCI Europe and used without further purification. 5,5′-dicyano-2,2′-bipyridine was synthesized following the procedure described in the literature [48].

### 3.2. Synthesis of Bipy-CTFs and Ru^III^@bipy-CTF Materials

The preparation of the bipy-CTF material was achieved following the standard procedure described in the literature applying the typical ionothermal conditions [32]. Typically, a glass ampule was filled with 5,5′-dicyano-2,2′-bipyridine (100 mg, 0.48 mmol) and ZnCl_2_ (332 mg, 2.40 mmol) in a glovebox. The ampule was flame-sealed under vacuum and placed in an oven at 400 °C for 48 h with a heating rate of 60 °C/h. After cooling to room temperature, the ampule was opened and the black-colored solid was stirred in 120 mL H_2_O overnight at 60 °C, filtered and washed with H_2_O and acetone. The solid was then stirred at 120 °C in 1 M HCl (150 mL) overnight, filtered, and subsequently washed with 1 M HCl (3 × 75 mL), H_2_O (15 × 75 mL), THF (3 × 75 mL), and acetone (3 × 75 mL). Finally, the powder was dried under vacuum overnight at 90 °C (Found for bipy-CTF: C, 58.92; H, 3.51; N, 20.27%).

The (Ru(acac)_2_(CH_3_CN)_2_)PF_6_ complex was prepared according to the literature method [49]. For this, (Ru(acac)_3_) (400 mg, 1 mmol) was dissolved in 30 mL of CH_3_CN and to this solution, 10 mL of a 1% H_2_SO_4_/CH_3_CN solution was slowly added while stirring. The solution was stirred at room temperature until the wine-red solution turned deep blue (approx. 12 h). Next, the solution was concentrated to 3 mL by evaporating the solvent. NH_4_PF_6_ (0.5 g, 3 mmol) in 5 mL of cold water was added to the deep-blue solution. The resulting deep-blue precipitate was collected by filtration, washed with cold water and n-hexane and dried under vacuum.

The post-modification of the bipy-CTF was performed as follows: (Ru(acac)_2_(CH_3_CN)_2_)PF_6_ (13.6 mg, 0.026 mmol) was added to 4 mL dry toluene. Afterward, 120 mg bipy-CTF material was added and stirred for 48 h at 80 °C. The prepared material was stirred in CH_3_CN for 24 h to remove the unreacted residue of the Ru complex. Then, the modified material was filtered, and washed thoroughly with CH_3_CN and acetone, followed by drying under vacuum overnight (Found for Ru^III^@bipy-CTF, C, 59.6; H, 3.2; N, 15.7%).

### 3.3. Catalytic Reactions

The procedure used to perform the tandem reaction is as follows: The oxidation of benzyl alcohol was carried out in a 20 mL Schlenk tube. During a typical catalytic test, the catalyst (1 mol % Ru), Cs_2_CO_3_ (0.4 mmol, placed in a porous membrane) as base, benzyl alcohol (0.33 mmol), dodecane as internal standard (0.33 mmol) and toluene (500 μL) were added to the Schlenk tube. The tube was purged with pure oxygen, sealed and heated to 100 °C for 12 h. Samples were withdrawn after 12 h. Upon cooling to room temperature and dilution with solvent, the samples were analyzed using a gas chromatograph. Hereafter, the porous membrane containing Cs_2_CO_3_ was removed and nitrile substrates (0.33 mmol) were added to the previous reaction mixture. The tube was sealed without purging oxygen. The reaction was heated from room temperature to 70 °C for an additional 1 h. Upon cooling to room temperature and dilution with toluene, the samples were analyzed using a gas chromatograph. After each catalytic run, the catalyst was recovered by filtration and washed with toluene, water, and acetone. The catalyst was then used directly in the subsequent runs. Conversion, selectivity and yield are calculated through Equations (S1)–(S3), respectively (see section catalytic reactions in SI, Figure S2).

## 4. Conclusions

In conclusion, an efficient heterogeneous catalyst is developed by applying a highly N-rich covalent triazine framework. The presence of bipyridine docking sites within the CTF provides excellent anchoring centers for the (Ru(acac)_2_(CH_3_CN)_2_)PF_6_ complex. The potential application of the obtained Ru^III^@bipy-CTF catalyst was studied in the selective tandem aerobic oxidation-Knoevenagel condensation reaction. The catalyst showed a very high conversion of various benzyl alcohol derivatives (80–99%) with full selectivity towards the corresponding α, β-unsaturated nitriles using O_2_ as the sole oxidant. The N-rich functionalities not only act as basic sites for Knoevenagel condensation reaction but also promote the aerobic oxidation of alcohols through oxygen activation. The obtained results revealed the high catalytic performance of the Ru^III^@bipy-CTF catalyst, exceeding its homogeneous counterpart. To the best of our knowledge, this is one of the rare reports on the application of Ru-based catalysts for tandem oxidation catalysis using O_2_ as the sole oxidant.

## Figures and Tables

**Table 1:** Elemental analysis of the pristine and modified CTF materials.

Sample	C ^a^ (wt.%)	N ^a^ (wt.%)	C/N	Ru ^b^ (mmol g^−1^)
bipy-CTF	58.92	20.27	2.9	-
Ru^III^@bipy-CTF	59.6	15.7	3.8	0.15

^a^ Determined by elemental analysis. ^b^ Determined by ICP-OES analysis.

**Table 2:** Catalytic performance of different catalysts in the oxidation of benzyl alcohol.

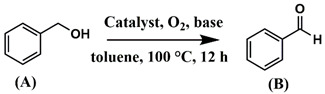				
Entry	Catalyst	Base	Conversion (%)	TON ^a^
1	Ru^III^@bipy-CTF	No base	37	37
2	Ru^III^@bipy-CTF	Na_2_CO_3_	41	41
3	Ru^III^@bipy-CTF	K_2_CO_3_	78	78
4	Ru^III^@bipy-CTF	Cs_2_CO_3_	99	99
5	Ru^III^@bipy-CTF ^b^	Cs_2_CO_3_	64	267
6	No catalyst	Cs_2_CO_3_	<1	-
7	Ru^III^@bipy-CTF ^c^	Cs_2_CO_3_	3	3
8	[Ru(acac)_2_(CH_3_CN)_2_]PF_6_	Cs_2_CO_3_	54	54
9	bipy-CTF ^d^	Cs_2_CO_3_	39	39

Reaction conditions: 1 mol% catalyst (based on Ru, obtained from ICP-OES analysis), 0.33 mmol benzyl alcohol, 0.4 mmol base, 500 μL toluene, O_2_, 100 °C, 12 h. ^a^ mmol of product formed per mmol of Ru in the catalyst. ^b^ 0.24 mol% catalyst was used. ^c^ Under Ar atmosphere. ^d^ 17 mg catalyst was used. All catalysts displayed >99% selectivity toward benzaldehyde.

**Table 3:** Catalytic performance of Ru^III^@bipy-CTF catalyst in tandem aerobic oxidation-Knoevenagel condensation reaction.

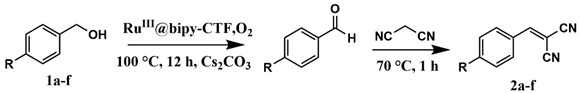			
Substrate	Product	Conversion of 1a–f (%)	Yield of 2a–f (%)
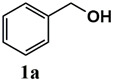	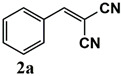	99	99
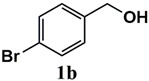	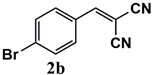	99	99
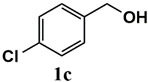	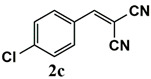	99	99
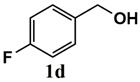	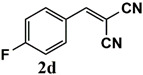	97	97
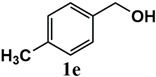	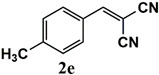	99	99
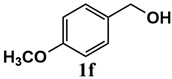	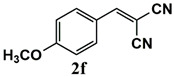	80	80

Reaction conditions: 1 mol% catalyst (based on Ru, obtained from ICP-OES analysis), 0.33 mmol alcohol, 0.4 mmol Cs_2_CO_3_, 500 μL toluene, 0.33 mmol malononitrile, O_2_, 100 °C, 12 h (1st step) and 70 °C, 1 h (2nd step). All substrates displayed >99% selectivity toward the corresponding product.

**Table 4:** Comparison of the Ru^III^@bipy-CTF catalyst with other heterogeneous catalysts for selective tandem oxidation-Knoevenagel condensation reaction.

Entry	Catalyst	Oxidant/Temp. (°C)	Time (h) ^a^	Conv./Yield (%)	Ref
1	Au@Cu(II)-MOF	Air/110	15 + 7	99/99	[42]
2	Au@MIL-53(NH_2_)	O_2_/100	13	99/99	[43]
3	UoB-2 (Ni-MOF)	TBHP/65	1.5	94	[44]
4	Cu_3_TATAT-3 MOF	O_2_, TEMPO/75	12	95/95	[45]
5	Pd/COF-TaPa-Py	O_2_/80	4 + 1.5	98/98	[46]
6	5CoOx/tri-g-C_3_N_4_	O_2_/80	6	96.4/96.4	[47]
7	Ru^III^@bipy-CTF	O_2_/100	12 + 1	99/99	This work

^a^ “m + n” refers to a step-by-step reaction without separation of benzaldehyde as the intermediate.

## Data Availability

The data and compounds presented in this study are available on request from the corresponding authors.

## Supplementary Materials

The following are available online, Table S1: Various control experiments to obtain insights into the reaction mechanism for the oxidation of benzyl alcohol, Figure S1: Proposed mechanism for bipy-CTF-catalyzed aerobic oxidation of benzyl alcohol, Figure S2: The GC-MS spectra of the α, β-unsaturated nitriles. Equation (S1): Conversion calculation of catalytic reaction. Equation (S2): yield calculation of catalytic reaction. Equation (S3): selectivity calculation of catalytic reaction.
