# Supplementary material for: A Ru-Complex Tethered to a N-Rich Covalent Triazine Framework for Tandem Aerobic Oxidation-Knoevenagel Condensation Reactions
Source: Molecules. 2021 Feb 5;26(4):838. doi: 10.3390/molecules26040838 (PMC7914989; doi:10.3390/molecules26040838)
Supplement: Supplementary file 1 [file molecules-26-00838-s001.pdf]

Supplementary Materials for

A Ru-complex tethered to a N-Rich Covalent Triazine Framework for Tandem Aerobic Oxidation–Knoevenagel Condensation Reactions

Geert Watson†, Parviz Gohari Derakhshandeh†, Sara Abednatanzi*, Johannes Schmidt, Karen Leus, Pascal Van Der Voort*

*Corresponding authors Email: Pascal.VanDerVoort@UGent.be; Sara.Abednatanzi@UGent.be

This PDF file includes:

Details of the catalytic reactions, mechanistic studies, Figures S1-S2, Equations S1-S3 and Tables S1.

Table S1. Various control experiments to obtain insights into the reaction mechanism for the oxidation of benzyl alcohol.

Figure S1. Proposed mechanism for bipy-CTF-catalyzed aerobic oxidation of benzyl alcohol.

Figure S2. The GC-MS spectra of the α, β-unsaturated nitriles.

1. Mechanistic studies

To obtain an insight of the function of the bipy-CTF material in oxidation catalysis, a set of control experiments was performed (Table S1). In this regard, a control experiment was performed using the pristine bipy-CTF material. A conversion of 39% was obtained using the pristine bipy-CTF as the catalyst in the presence of Cs_2CO_3 as the base. (Table S1, entry 1). To obtain more insight into the role of the base, the influence of different bases was investigated. The obtained results are outlined in Table S1 below. As can be observed, the reaction without any basic additive resulted in no conversion of benzyl alcohol (Table S1, entry 2). However, the addition of a variety of bases, such as Na_2CO_3, K_2CO_3, and Cs_2CO_3, improved the catalytic activity, with the highest activity obtained using Cs_2CO_3. In general, carboxylates with bigger counter cations are more dissociated in aprotic solvents and consequently more reactive. Similar trend is observed for the Ru^{III}@bipy-CTF catalyst (Table 2, entries 1-4 of the manuscript). We believe that the applied base can facilitate the deprotonation of benzyl alcohol and boosts the catalytic activity. The conversion of benzyl alcohol towards benzaldehyde dramatically lowered to 3% under Ar atmosphere, proving the essential role of oxygen as the oxidant (Table S1, entry 5). Another control experiment was done using *p*-benzoquinone (Table S1, entry 7). The addition of *p*-benzoquinone as superoxide ($^{\bullet}O_2^-$) scavenger into the reaction system containing bipy-CTF as the catalyst completely suppressed the oxidation of benzyl alcohol (conversion < 1%). This observation indicates that bipy-CTF activates molecular oxygen to superoxide, which acts as oxidant species in the catalytic cycle.

Table S1. Various control experiments to obtain insights into the reaction mechanism for the oxidation of benzyl alcohol.

Entry	Catalyst	base	Conversion (%)
1	bipy-CTF	Cs_2CO_3	39
2	bipy-CTF	No base	3
3	bipy-CTF	K_2CO_3	18
4	bipy-CTF	Na_2CO_3	10
5[a]	bipy-CTF	Cs_2CO_3	3
6[b]	bipy-CTF	Cs_2CO_3	14
7[c]	bipy-CTF	Cs_2CO_3	<1

Reaction conditions: 17 mg bipy-CTF, 0.33 mmol benzyl alcohol, 0.4 mmol base, 500 µl toluene, O_2, 100 °C, 12 h. [a]Under Ar atmosphere. [b]Reaction was done at 50°C. [c]*p*-benzoquinone (0.33 mmol) was added.

So far, many experimental and theoretical investigations have shown the applicability of N-doped carbon-based materials for adsorption and activation of molecular O_2.[1-2] Based on our observations and previous reports,[3-4] we propose that the oxygen activation occurs on the surface of bipy-CTF and the generated superoxide reacts with benzyl alcohol to generate $PhCH_2O^-$ and subsequently $PhCH_2O^{\bullet}$ and benzaldehyde. Although the exact mechanism on the aerobic oxidation using $[Ru(acac)_2(CH_3CN)_2]PF_6$ complex has been remained unknown in the previous reports, it can be expected that the generated $PhCH_2O^-$ on the surface of bipy-CTF reacts with the Ru centers in the reaction system. The obtained results thus corroborate each other, unveiling the promotional impact of bipy-CTF in activating oxygen and benzyl alcohol in our reaction system. A reaction mechanism is proposed based on the obtained results and previous studie[3-4], as shown in Figure S1.

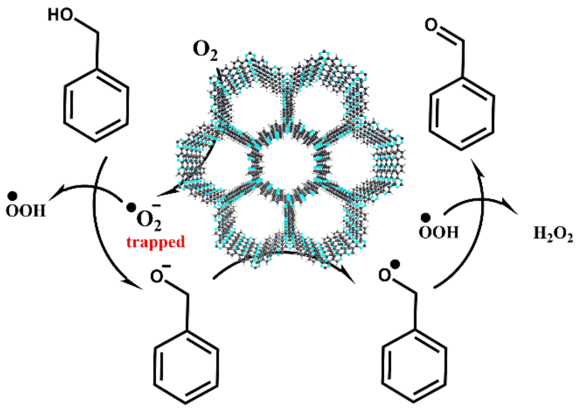

Figure S1. Proposed mechanism for bipy-CTF-catalyzed aerobic oxidation of benzyl alcohol.

2. Catalytic reactions

Conversion, yield and selectivity are calculated through equations S1, S2 and S3 respectively. Dodecane was used as the internal standard. The conversion, yield and selectivity are calculated based on the observed peak area using a TRACE GC × GC (Thermo, Interscience) coupled to a TEMPUS TOFMS detector (Thermo, Interscience). The pure starting material (alcohols) and products (α, β-unsaturated nitriles) were injected to the GC as the reference peaks.

$$(S1)\quad Conversion = 1 - \left(\frac{Area\ of\ IS1}{Area\ of\ S1} \times \frac{Area\ of\ S2}{Area\ of\ IS2}\right) \times 100$$

$$(S2)\quad Yield = \frac{mmol\ of\ S1 - mmol\ of\ S2}{mmol\ of\ S1} \times 100$$

$$(S3)\quad Selectivity = \frac{Area\ of\ Px}{Area\ of\ all\ products} \times 100$$

IS1= Internal standard at time zero; S1= Substrate at time zero; S2= Substrate at time X; IS2= Internal standard at time X; PX= Product X.

The GC-MS spectra of the α, β-unsaturated nitriles are presented below.

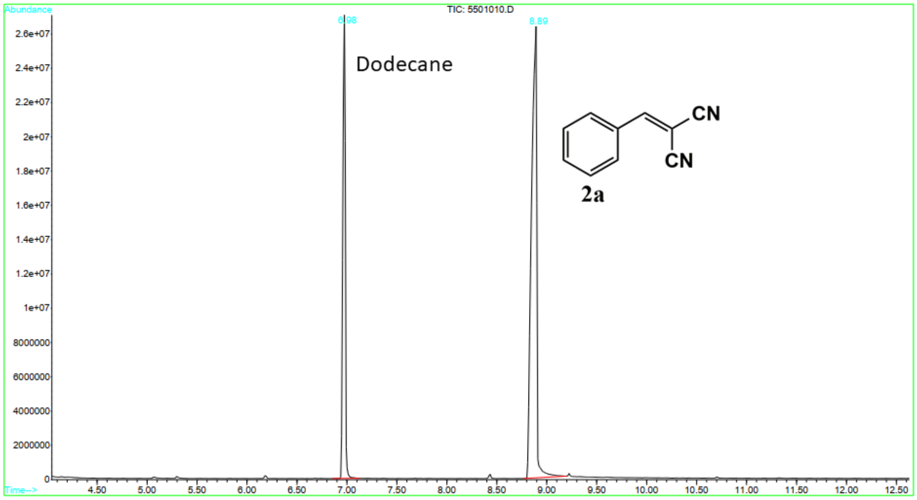
TIC: 5501010.D
Dodecane
2a
CN
CN

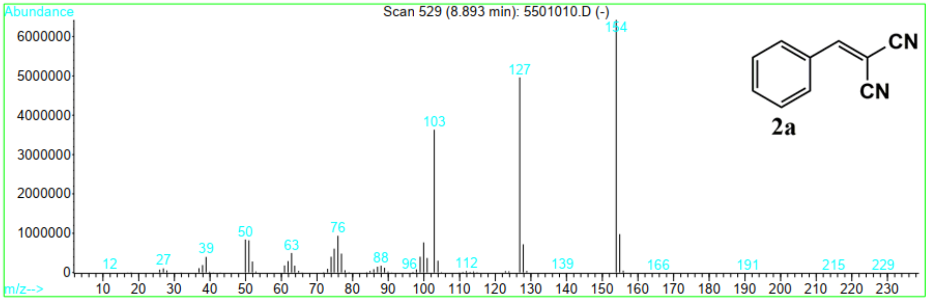
Scan 529 (8.893 min): 5501010.D (-)
2a
CN
CN

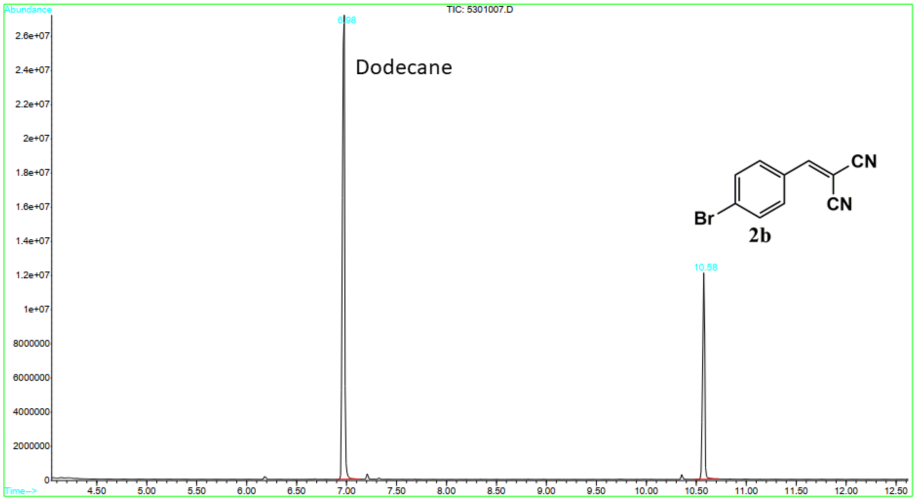
TIC: 5301007.D
Dodecane
2b
Br
CN
CN

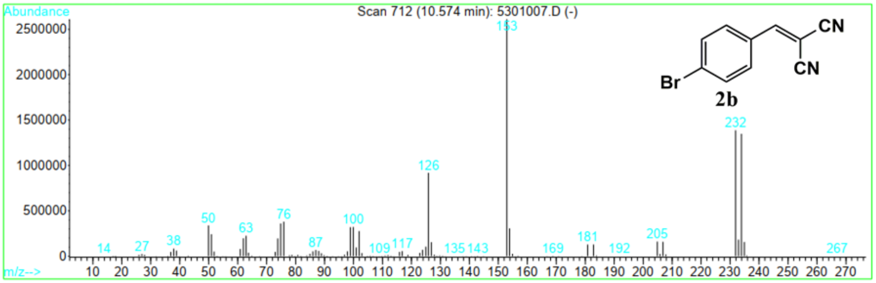
Abundance
Scan 712 (10.574 min): 5301007.D (-)
2b
Br
CN
CN
m/z-->
153
232
126
50
63
76
87
100
205
181
267

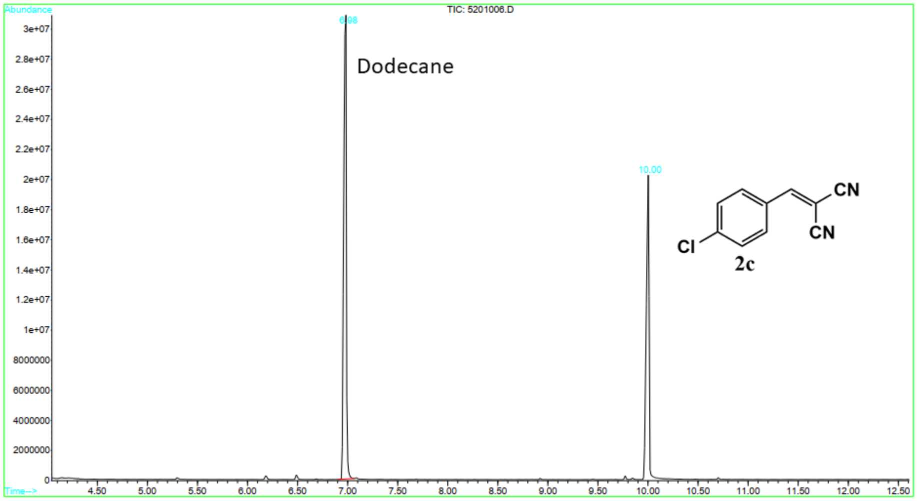
Abundance
TIC: 5201006.D
Dodecane
10.00
Cl
CN
CN
2c
Time-->
4.50
5.00
5.50
6.00
6.50
7.00
7.50
8.00
8.50
9.00
9.50
10.00
10.50
11.00
11.50
12.00
12.50

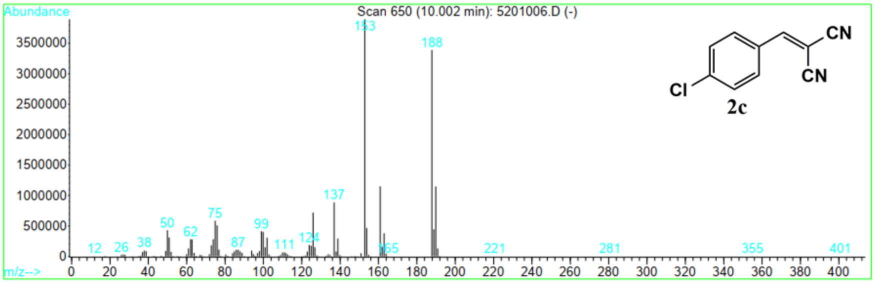
Abundance
Scan 650 (10.002 min): 5201006.D (-)
Cl
CN
CN
2c
m/z-->
153
188
137
50
62
75
87
99
111
221
281
355
401

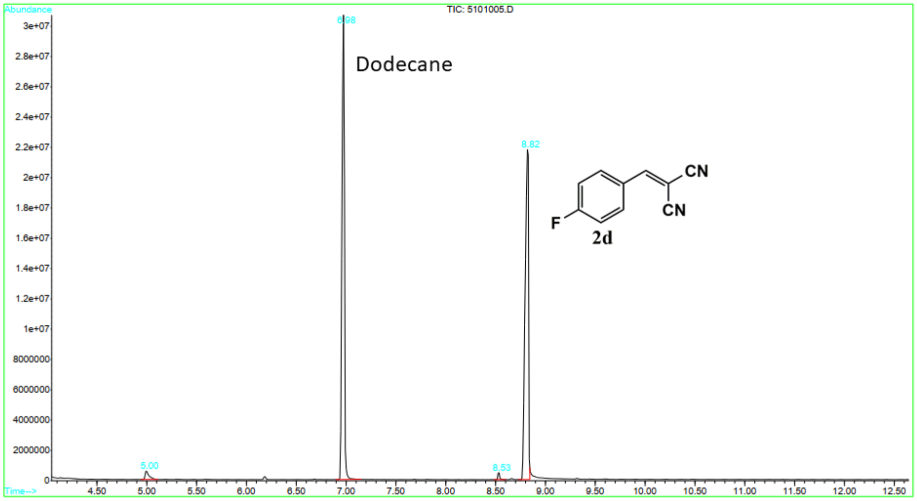
TIC: 5101005.D
Dodecane
2d

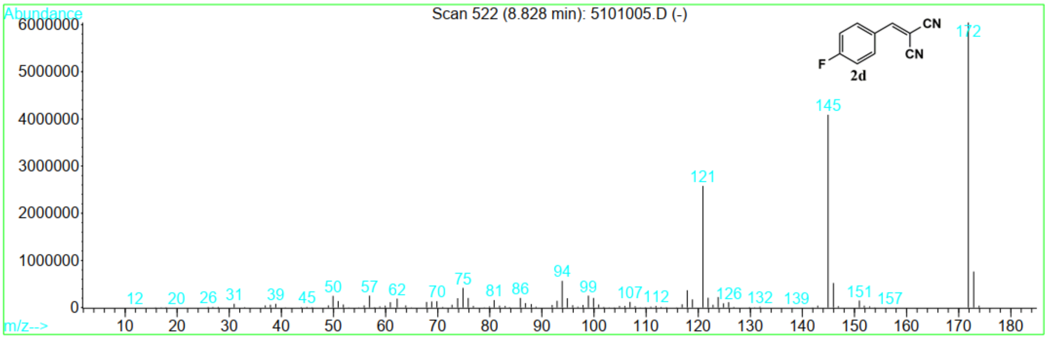
Scan 522 (8.828 min): 5101005.D (-)
2d

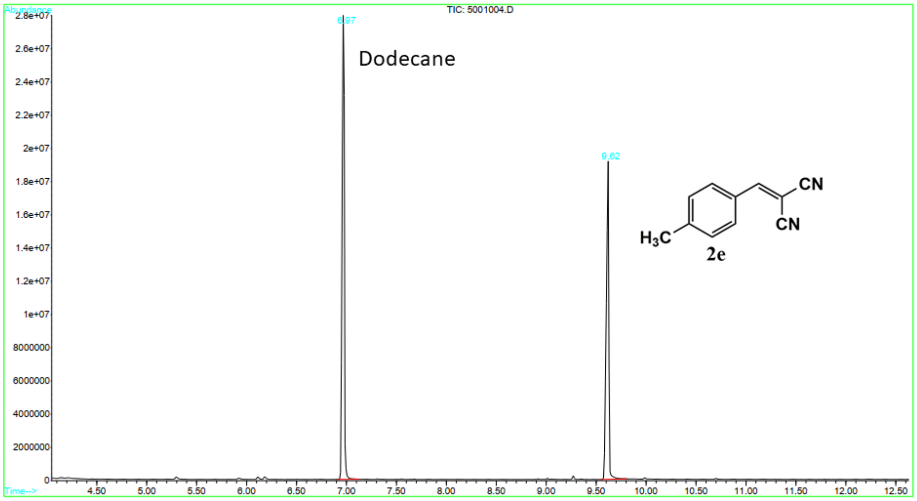
TIC: 5001004.D
Dodecane
2e

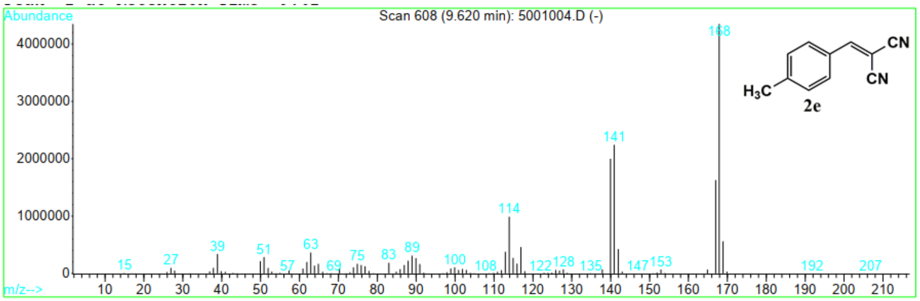

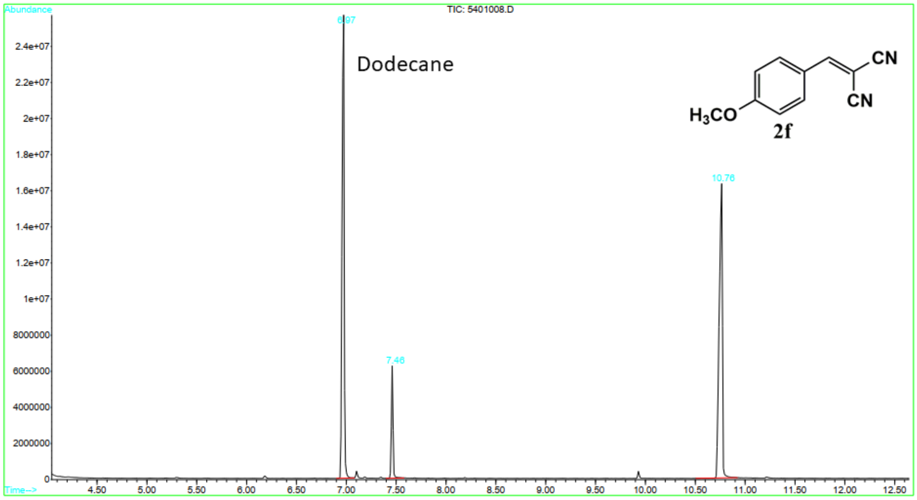

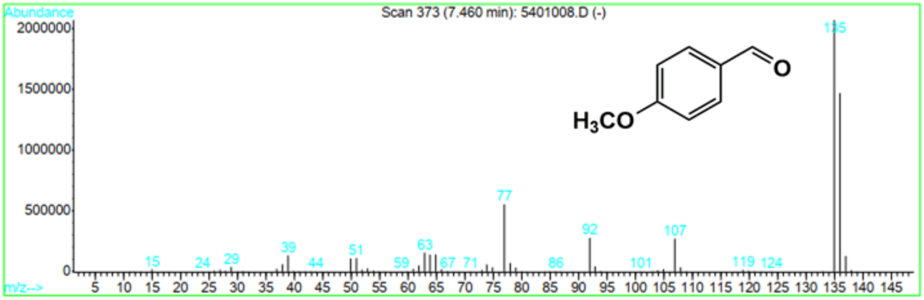

Peak 3 at Retention Time 10.76

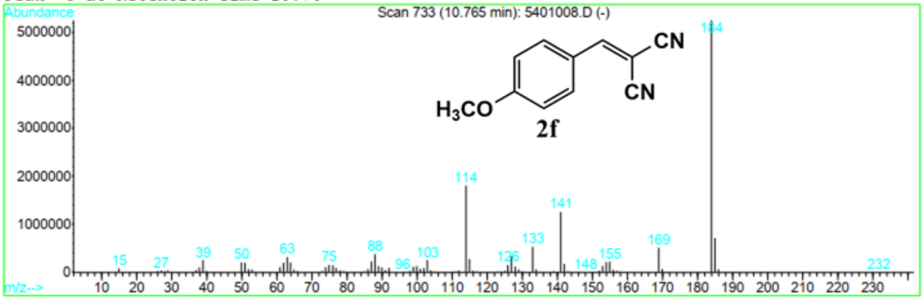

Figure S2. The GC-MS spectra of the α, β-unsaturated nitriles.

References:

[1] Watanabe, H.; Asano, S.; Fujita, S.; Yoshida, H.; Arai, M., Nitrogen-Doped, Metal-Free Activated Carbon Catalysts for Aerobic Oxidation of Alcohols. *Acs Catal* **2015,** *5* (5), 2886-2894.

[2] Eisenberg, D; Slot, T.K; Rothenberg, G.; Understanding oxygen activation on metal- and nitrogen-codoped carbon catalysts. *ACS Catal* **2018,** 8, 8618–8629.

[3] Abednatanzi, S.; Derakhshandeh, P. G.; Leus, K.; Vrielinck, H.; Callens, F.; Schmidt, J.; Savateev, A.; Van der Voort, P., Metal-free activation of molecular oxygen by covalent triazine frameworks for selective aerobic oxidation. *Sci Adv* **2020,** *6* (14).

[4] Abednatanzi, S.; Derakhshandeh, P. G.; Tack, P.; Muniz-Miranda, F.; Liu, Y. Y.; Everaert, J.; Meledina, M.; Vanden Bussche, F.; Vincze, L.; Stevens, C. V.; Van Speybroeck, V.; Vrielinck, H.; Callens, F.; Leus, K.; Van Der Voort, P., Elucidating the promotional effect of a covalent triazine framework in aerobic oxidation. *Appl Catal B-Environ* **2020,** *269*.
